# Physical Activity in the Elderly and Frailty Syndrome: A Retrospective Study in Primary Care

**DOI:** 10.3390/medicines9100051

**Published:** 2022-10-11

**Authors:** Abrar-Ahmad Zulfiqar, Habib Habchi, Perla Habchi, Ibrahima Amadou Dembele, Emmanuel Andres

**Affiliations:** 1 Service de Médecine Interne, Diabète et Maladies Métaboliques de la Clinique Médicale B, Hôpitaux Universitaires de Strasbourg et Equipe EA 3072 “Mitochondrie, Stress Oxydant et Protection Musculaire”, Faculté de Médecine, Université de Strasbourg, 67000 Strasbourg, France; 2 Département de Médecine Générale, Université de Reims, 51100 Reims, France; 3 Anesthesiology, Aman Hospital, F Ring Rd, Zone 47, Building 412, Doha P.O. Box 8199, Qatar

**Keywords:** physical activity, frail elderly, Ricci–Gagnon questionnaire, general practice

## Abstract

Objectives: Physical activity carries numerous therapeutic benefits, and it is more effective when applied before the onset of symptoms. The objective of this study is to compare the correlation of the evaluation of physical activity carried out using the Ricci and Gagnon test and the frailty profile measured by the mSEGA scale in a population of patients consulting in general medicine. Methods: We conducted a retrospective study within a general practitioner clinic in Chaumont and Bologne (Haute-Marne department) during a 3-month period. Patients aged 65 years and up were screened for frailty using the modified SEGA (mSEGA) assessment, and physical activity was measured using the Ricci–Gagnon questionnaire. Results: A total of 44 patients were selected, with a slightly female predominance (59.1%). Of these, 21 patients reported having worked in manual labor. Seven patients were found to be frail using the SEGAm assessment, while 10 (22.73%) patients had an inactive profile according the Ricci–Gagnon score. Malnutrition was detected in six patients (13.64%) using the MNA survey. Frailty as defined by the mSEGA scale had no statistical correlation (*p* = 0.68) with the Ricci–Gagnon score. A Ricci–Gagnon inactive profile showed statistical correlations with fall indicators (unipedal balance test, *p* = 0.014) and malnutrition scores using the MNA (*p* = 0.0057) as well as with the Charlson Comorbidity Index (*p* = 0.027). Conclusion: A systematic survey of the elderly by a general practitioner implementing a regular and suitable physical activity regimen would allow a better screening of frailty, minimizing its complications.

## 1. Introduction

It is known and recognized that physical activity is beneficial to the elderly. The most active elderly people have a lower all-cause mortality rate (coronary heart disease, arterial hypertension, stroke, type 2 diabetes, colon and breast cancer) [1], a greater cardio-respiratory and muscular capacity, healthier body mass by increasing the ratio of lean mass to fat mass [2]; biological markers indicating greater prevention of cardiovascular disease, type 2 diabetes and strengthening of bone quality [3,4]; better functional health, less risk of falling, and better cognitive functions [5]; and they are less likely to be moderately or severely limited in their functioning and to see their social role diminish [3].

All elderly subjects are confronted with the relatively recent concept of frailty, for which it is difficult to find a consensual definition since it is a dynamic, evolving and multidimensional concept comprising factors that are physical, physiological, biological, social and environmental. All the authors agree that it is a preponderant indicator of morbidity and mortality. Some of these factors are reversible, particularly through the practice of aerobic physical activity, making their detection and prevention a major public health issue. According to the High Authority for Health (HAS), there are two models of frailty in the literature:

- The phenotypic model developed by Linda Fried in the 1990s, based on so-called functional frailty with, at the heart of this approach, the concept of sarcopenia [6]. This category of frailty represents 30 to 40% of people aged over 65 worldwide [7].
- The clinical model, developed by the Canadian team at Rockwood, based on multidimensional frailty, taking into account physical, psychological and social factors, and enabling researchers to obtain a frailty index [8].

Early detection of these potentially reversible factors through multidisciplinary care, and appropriate physical activity in particular, is a real public health issue that could reduce certain health costs related to aging.

Because of their privileged relationship and repeated contact with the elderly, the general practitioner is the first point of contact for the early identification of frailty in elderly people. It is therefore possible to imagine the implementation of strategies aimed at reversing the state of frailty—promoting the practice of a physical activity, for example, adapted to the condition and comorbidities of the elderly subject, and intended to improve their quality of life. As the French population faces inevitable aging, it is judicious and essential to ensure optimal monitoring of this aging, primarily through the general practitioner, to improve any cases of fragility, regardless of severity. This key prevention role has multiple facets of possible action within a general practice, with one major component lying within the practice of regular, adapted, reassuring and progressive physical activity, according to the criteria of the French Society of Sport-Health (SF2S). Health insurance in France promotes physical activity in all subjects, including the elderly. Thus, a campaign of screening and quantification of physical activity is carried out with awareness raising among general practitioners. The Ricci–Gagnon questionnaire, available on the ameli.fr website, is thus proposed, despite a lack of validity and reproducibility. In fact, no references have been found in the literature regarding the use of the Ricci–Gagnon questionnaire in elderly subjects in primary care and its link to frailty syndrome. This is the first time such a study has been carried out.

Thus, in this study, we sought to identify the presence of an association between the practice of physical activity measured by Ricci–Gagnon questionnaire and the profile of frailty according to the scale of the modified Short Emergency Geriatric Assessment (mSEGA), in patients aged 65 and over, and consulting a general practice. Secondarily, we studied the impact of this association on several frailty factors (nutrition, cognition, and comorbidities).

## 2. Patients and Methods

### 2.1. Type of Study

This was a retrospective study carried out in a general practice in Chaumont and Bologna, (Region Grand Est) France, spanning from 1 February 2016 to 30 April 2016 inclusive.

### 2.2. Inclusion and Exclusion Criteria

The inclusion criteria were an age of 65 years or older at the time of the general medicine consultation. We excluded patients consulting for an acute pathology and requiring hospitalization.

### 2.3. Data Collected

For each patient, data were obtained from the patients’ electronic medical record:

- Sex, age, reason for consultation, medical and surgical history, marital status, professional status during their working life, presence or absence of addictive behaviors.
- Frailty was measured by the mSEGA grid. For the mSEGA score [9], we separated the patients into three groups: non-frail patients if the score was less than 8, frail patients if the score was between 8 and 11, and very frail patients if the score was greater than 11.
- The biometric level was appreciated by the use of arm circumference (normal value > 21 cm), calf circumference (normal value >31 cm), height (using heel/knee height when standing measurements were impossible), and weight. From these values, we were able to calculate the Body Mass Index (BMI) from the formula: weight (in kg)/(size^2^) (in m), and whose interpretation followed the values established by HAS (Haute Autorité des Soins): normal BMI if < or = 24.9 kg/m^2^. We also carried out the Mini Nutritional Assessment (MNA), which enabled us to define whether the patients were: not malnourished with a score > 24, at risk of malnutrition with a score between 17 and 24 or malnourished with a score < 17.
- Data from the Comprehensive Geriatric Assessment (CGA) were also collected, with the single-leg support test, which was defined as positive if the single-leg support was > or equal to 5 s. Dependence was assessed using the Katz and Lawton scales (ADL and IADL). Memory disorders according to the Mini Mental State Examination (MMSE) score were found in the medical file.
- The sedentary lifestyle and physical activity test was studied using the Ricci–Gagnon (R&G) scale, with 2 distinct groups: inactive (score < 18) and active (score ≥ 18).
- From a biological point of view, we noted the following values dating from less than 6 months within the clinic visit: albumin (hypoalbuminemia was defined by albuminemia < 35 g/L), creatinine (kidney failure was defined by a glomerular filtration rate (GFR) < 60 mL/min/1.73 m^2^), hemoglobin (Hb) (anemia was defined by hemoglobinemia < 12 g/dL), vitamin D (a vitamin D deficiency corresponded to a level < 30 ng/mL).

### 2.4. Statistical Analysis

Data were analyzed with SAS software version 9.4 (SAS Institute Inc., Cary, NC, USA). The qualitative variables were described in the form of counts and percentages, with quantitative variables in the form of mean and standard deviation. Cross-sortings were carried out using the Chi-square test. The significance level of the statistical tests carried out was set at *p* < 0.05 for all tests.

### 2.5. Administrative Elements:

From a regulatory standpoint, informed consent was obtained from all patients included in this study. From an ethical and regulatory point of view, the study obtained authorization from the National Commission for Computing and Liberties (CNIL: declaration n° 1932408).

## 3. Results

**Figure 1:**
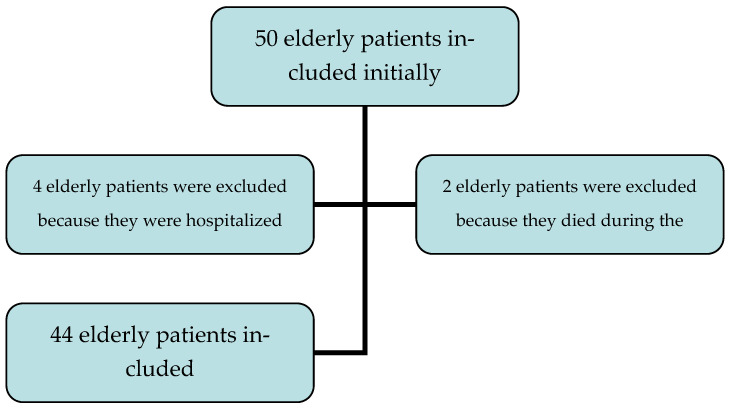
Patient Inclusion Flowchart.

A total of 44 patients were included between 1 February and 30 April 2016. See the flowchart (Figure 1). Of these, 26 patients were female (59.1%), with a sex ratio F/M of 1.4. There were four patients over the age of 85 (9.09%). See Table 1 and Table 2 for more details.

This is a sample of 44 elderly subjects. Within our study, we did not find a correlation between frailty according to the mSEGA score and physical activity according to Ricci–Gagnon (*p* = 0.68).

We studied physical activity according to the Ricci–Gagnon score and found a significant link between inactivity according to the Ricci–Gagnon scale (score < 18), and the pathological monopodal support test (*p* = 0.014), undernutrition according to the MNA (*p* = 0.0057) and the Charlson comorbidity score (*p* = 0.027) and a slightly significant trend with age, which was most likely linked to a lack of potency (*p* = 0.06). We did not find any significant correlation with the team sport item (*p* = 0.15), undernutrition according to BMI (*p* = 0.68), undernutrition according to albumin (*p* = 0.3), and vitamin D deficiency (*p* = 0.61). See Table 3.

## 4. Discussion

In our study, we evaluated physical activity according to the Ricci–Gagnon questionnaire. This questionnaire was developed in Canada to promote physical activity in a hydroelectric company in Quebec [10]. To our knowledge, there have been no previous studies looking for a correlation between the level of physical activity assessed by this score and the presence of frailty. We found a study published in 2011 by Pavy et al. [10] focused on cardiac rehabilitation in coronary patients, which demonstrated a significant correlation between the Ricci–Gagnon score and validated objective measures, including the exercise test (correlation coefficient r = 0.29, *p* < 0.0001) and the 6-min walk test (TM6, r = 0.40, *p* < 0.0001). This testifies to an objectivity concerning the Ricci–Gagnon score compared to other validated methods.

Our results show the absence of correlation between frailty according to the modified SEGA scale [9,11] and physical activity according to Ricci–Gagnon, which is certainly due to a lack of scale. Our work could serve as a pilot study for other trials of considerable size in order to establish a significant correlation between these two parameters, allowing the validation of this simple and rapid questionnaire, and its use by general practitioners to detect and monitor frailty in elderly patients.

We chose the Ricci–Gagnon questionnaire for its simplicity and short duration—important details in general medicine consultation—but also to identify statistical trends with clinical entities such as undernutrition or frailty, which have not been established before. This makes our work unique in its kind and paves the way for other specific research to validate this little-studied score. Although the Ricci–Gagnon questionnaire has not been validated to date, according to the data in the literature, our work is innovative in the sense that we have studied the link between the physical activity measured by this questionnaire and frailty syndrome, which is a first. Additional large-scale studies are needed to validate the Ricci–Gagnon questionnaire and study its links with frailty syndrome according to several frailty scales. In the literature, other physical activity scales are used, including the International Physical Activity Questionnaire (IPAQ). Created in 2003, it is aimed at all subjects over the age of 15 [12]. This questionnaire assesses overall physical activity and the level of sedentary lifestyle over the prior seven days. The questionnaire focuses on the practice of intense to moderate activities, walking, as well as time spent sitting (sedentary), whether during leisure activities, at work, in daily life, or even during transport. Many articles report a significant link between physical activity and its level measured by IPAQ and the determinants of frailty syndrome [13,14].

Using the SEGA scale in our present study, we were not able to establish a significant correlation with the level of physical activity according to Ricci–Gagnon (*p* = 0.68) for lack of sustained power. It nevertheless remains an objective and essential identification tool in the search for a state of frailty. The term “sarcopenia” was first described by Rosenberg in 1989, and it was further developed by Baumgartner in 1998 [15] as an isolated decrease in muscle mass. Etymologically, it derives from the Greek “Sarx” (flesh) and “Penia” (loss). A consensual definition was put forward by the European Working Group on Sarcopenia in Older People (EWGSOP) in 2010 [16], and it includes three factors: decreased muscle mass (assessed by calf circumference < 31 cm), in conjunction with decreased muscle strength (measured by grip strength, using a dynamometer) and/or decreased muscular performance (measured by several tools, including a walking speed over 4 m < 0.8 m/s) [17]. Sarcopenia constitutes a fundamental element of frailty according to Bauer et al. [18], and it lies at the heart of the phenotypic approach to frailty proposed by Fried. It is associated with an increased risk of dependence and all-cause mortality as well as higher healthcare costs [19].

As a result, physical activity plays a major role in the prevention of sarcopenia, and consequently, frailty. In a recent study of 2309 people aged over 65, Mijnarends et al. [20] found a positive correlation between increased moderate or intense physical activity (MVAP) and improved aspects of sarcopenia (mass, strength, and muscle performance). They concluded that this reduced the incidence of sarcopenia (OR = 0.64, 95% CI 0.45–0.91).

Another meta-analysis [21] examined the overall benefits of resistance physical activity on sarcopenia (evaluating lean body mass). The results obtained after analyzing 49 studies showed a positive effect of resistance physical activity on lean mass gain and therefore on the reduction in sarcopenia.

Thus, the contribution of physical activity is essential to preventing and delaying the onset of sarcopenia. Although few practical recommendations exist on the nature and duration of physical activity in the elderly, this therapy, coupled with a balanced and appropriate diet, remains essential [22].

Interestingly, there are several moderators of this positive influence of physical activity on the overall health of older people, including food hygiene. Several studies have highlighted the superior effectiveness that a combination of physical activity and nutritional supplementation has on cognitive performance [23,24], as well as muscle performance and body composition in elderly subjects [25,26]. Food hygiene not only concerns protein intake but also includes several nutritional categories (protein, lipid, vitamin intake (C, E, D), minerals and antioxidants) for the prevention of sarcopenia and the consequential onset of fragility [27].

In our study, we noted the existence of a significant correlation between the inactive profile according to Ricci–Gagnon and the presence of malnutrition assessed by the MNA questionnaire (*p* = 0.005 and *p* = 0.02, respectively). The intertwined effects of physical activity and undernutrition seem to act in two directions in the elderly: an optimal nutritional state would contribute to the improvement and maintenance of regular physical activity, which itself would influence the nutritional profile by modulating the body’s metabolism.

Therefore, nutritional intervention is just as essential against the development of frailty, and it must be considered in association with the practice of physical activity for optimal care in the elderly, with or without malnutrition [28].

The Charlson score is a validated tool in elderly subjects [29]. It is used to calculate and assess the importance of associated comorbidities. In this cohort study, we established a significant link between the presence of an inactive profile according to Ricci–Gagnon and a pejorative morbidity score calculated by Charlson (*p* = 0.027). This is consistent with publications in the scientific literature, which demonstrate a reduction in comorbidities following the practice of physical activity [30].

### Limits

We must point out the presence of several limitations to this study. The most important of these concerns the sample size, which hinders obtaining statistically significant results.

The choice of questionnaire used to assess physical activity also raises some questions; we chose the Ricci–Gagnon score, among a whole panel of questionnaires, for its simplicity and practicability in the elderly. However, the Ricci–Gagnon score has not been validated, and it remains little described in the international literature. In a 2012 critical review comparing the different physical activity questionnaires administered to the French population, Vuillemin et al. did not evaluate the Ricci–Gagnon test and specified: “not having sufficient elements to judge the quality of this questionnaire” [31].

We specify, all the same, that the questionnaire is part of the Bilan Médico-Sportif (BMS) proposed in the Sport-Health course of the Sports Health Well-Being Network (RSSBE) of Champagne-Ardenne [32], and it is also proposed on the website mangerbouger.fr. It is therefore a tool used on a national scale in multiple medical fields.

## 5. Conclusions

According to our results, an evaluation of physical activity should be carried out in view of the established correlations. This would enable general practitioners to adapt the prescription of a physical activity to each elderly patient encountered.

In this context, the Ricci–Gagnon questionnaire seemed like an appropriate tool for this approach; it is simple and easy to complete, with a relatively short running time (close to 5 min), and it allows researchers to obtain a numerical score to classify patients.

By grouping these exposed elements, it becomes more realistic and possible to offer this geriatric screening consultation from a threshold age of 65 years and comprising:

- Geriatric screening focused on frailty;
- An evaluation of physical activity using the Ricci–Gagnon questionnaire.

This consultation would be interesting and essential for carrying out an adequate screening—all the more so if performed in a systematic way, at an annual frequency in a general practice. Larger-scale work should be carried out with the Ricci–Gagnon questionnaire in several general medicine practices.

## Figures and Tables

**Table 1:** Description of the sample population.

		*n* = 44
Sex, *n* (%)	Female	26 (59.1)
	Male	18 (40.9)
Age, m		75 (65–93)
Charlson, out of 24, m (sd)		2.8 (0–7)
Marital status		
Married		30 (68%)
Divorced		4 (9%)
Widowed		10 (23%)
Addictive behaviors		
Tobacco		16 (36.36%)
Alcohol		30 (68.18%)
Medical history		
Arthrosis		27 (61.4%)
Hypertension		22 (50%)
Obesity		14 (31.8%)
Pulmonary disease		12 (27.2%)
Heart deficiency (defined by left ventricular ejection fraction <50%)		10 (22.7%)
Diabetes		9 (20.45%)
Atrial fibrillation		8 (18.2%)
Neoplasms		6 (13.6%)
Prothesis		6 (13.6%)
Stroke		4 (9%)
Renal deficiency (defined by a glomerular filtration rate (GFR) <60 mL/min/1.73 m^2^)		4 (9%)
Cognitive disorders		3 (6.8%)
Nutritional status		
Undernourishment according to albumin		3 (8.82%)
Undernourishment according to Body Mass Index		11 (25%)
Undernourishment according to Mini Nutritional Assessment		6 (13.64%)
Biological measurement		
Anemia		1 (6.3%)
Vitamin D deficiency		34 (85%)
Frailty status		
Modified SEGA		7 (15.91)
Inactive Physical status		
According to Ricci–Gagnon scale		10 (22.73%)
Collective sport activity		6 (13.64%)

**Table 2:** Statistical analyses about nutritional, cognitive, frailty, biological and geriatric components.

Parameters	*n*	Mean	Standard Deviation	Median	Minimum	Maximum
Age (years)	44	75.0	7.1	73.5	65.0	93.0
Weight (kg)	44	72.5	13.6	70.5	47	117
Height (cm)	44	162.7	8.5	163	145	180
BMI (kg/m^2^)	44	27.4	5.1	26.2	20.8	43
Arm Circumference (cm)	44	31.6	3.8	31.5	23.5	43.5
Thigh Circumference (cm)	44	53.5	5.6	53.5	43.5	69.5
Calf Circumference (cm)	44	36.6	2.9	35.8	31	44.5
Abdominal Perimeter (cm)	44	101.6	11.7	101.5	81	138.5
Charlson	44	2.8	1.8	2	0	7
MMSE	44	25.4	3.3	26	18	30
mSEGA	44	5.3	2.9	5.0	1.0	12.0
IADL	44	6.7	1.6	7	1	8
ADL	44	5.8	0.4	6	4	6
Ricci Gagnon	44	22.6	6.3	22	9	34
Hb (g/dL) (missing data for one patient)	43	14	1.3	14.2	11.2	16.8
Glomerular filtration rate (mL/min/1.73 m^2^)	44	76.5	21.2	78.5	16	133
Albumin (g /L) (missing data for 10 patients)	34	38.4	3.2	38	31.3	45
Vitamin D (ng/mL) (missing data for 10 patients)	30	20.6	9	20	5	46
MNA	44	26.2	2.3	26.5	20	30

BMI: Body Mass Index; MMSE: Mini Mental State Examination; mSEGA: modified Short Evaluation Geriatric Assessment; IADL: Instrumental Activity of Daily Living; ADL: Activity of Daily Living; Hb: Hemoglobin; MNA: Mini Nutritional Assessment.

**Table 3:** Summary of correlations frailty syndrome and inactive profile according to Ricci–Gagnon.

**Parameter Correlation**	**Frailty According to mSEGA**
Inactive according to Ricci–Gagnon	*p* = 0.68
Pathological monopodal support test	*p* = 0.083
Collective sport	*p* = 0.25
Undernourishment BMI	*p* = 0.47
Undernourishment MNA	*p* = 0.21
Undernourishment Albumin	*p* = 0.002
Charlson score	*p* = 0.07
MMSE	*p* = 0.48
**Parameter Correlation**	**Inactive According to Ricci–Gagnon**
Pathological monopodal support test	*p* = 0.014
Age	*p* = 0.06
Undernourishment BMI	*p* = 0.68
Undernourishment MNA	*p* = 0.0057
Undernourishment Albumin	*p* = 0.3
Charlson score	*p* = 0.027
Vitamin D deficiency	*p* = 0.61

BMI: Body Mass Index; MNA: Mini Nutritional Assessment; MMSE: Mini Mental State Examination.

## Data Availability

The datasets used and/or analyzed during the current study are available from the corresponding author on reasonable request.
